# Reversible monolayer-to-crystalline phase transition in amphiphilic silsesquioxane at the air-water interface

**DOI:** 10.1038/srep08497

**Published:** 2015-02-17

**Authors:** R. Banerjee, M. K. Sanyal, M. K. Bera, A. Gibaud, B. Lin, M. Meron

**Affiliations:** 1 Surface Physics and Material Science Division, Saha Institute of Nuclear Physics, 1/AF Bidhannagar, Kolkata-700064, India; 2 LUNAM, IMMM, Faculté de Sciences, Université du Maine, UMR 6283 CNRS, Le Mans Cedex 9, 72085 France; 3 Center for Advanced Radiation Sources, The University of Chicago, Chicago, IL 60637, USA

## Abstract

We report on the counter intuitive reversible crystallisation of two-dimensional monolayer of Trisilanolisobutyl Polyhedral Oligomeric SilSesquioxane (TBPOSS) on water surface using synchrotron x-ray scattering measurements. Amphiphilic TBPOSS form rugged monolayers and Grazing Incidence X-ray Scattering (GIXS) measurements reveal that the in-plane inter-particle correlation peaks, characteristic of two-dimensional system, observed before transition is replaced by intense localized spots after transition. The measured x-ray scattering data of the non-equilibrium crystalline phase on the air-water interface could be explained with a model that assumes periodic stacking of the TBPOSS dimers. These crystalline stacking relaxes upon decompression and the TBPOSS layer retains its initial monolayer state. The existence of these crystals in compressed phase is confirmed by atomic force microscopy measurements by lifting the materials on a solid substrate.

It is believed that the lowest energy state in materials is crystalline as all the elements except helium and vast array of compounds equilibrate in crystalline state and once this state is achieved one requires substantial change in temperature and/or pressure to take a material into non-crystalline state. However, reversible crystallization in the tendons in the forearms of animals when stretched and/or dried^1^ and reversible structural changes of proteins in trans-membrane proton pumping mechanism of purple membrane (bacteriorhodopsin)^2^,^3^ have been observed. Reversible crystallization in polymers observed through small angle X-ray scattering^4^ and atomic force microscopy (AFM)^5^ have revealed that a material may remain in a non-equilibrium reversible state, trapped in a local minimum rather than in the equilibrium crystalline state which, once attained, is irreversible. Here we report reversible crystallization of two-dimensional monolayer of Trisilanolisobutyl Polyhedral Oligomeric SilSesquioxane (TBPOSS) on water surface and by repeating the reversible crystallization cycle one can form well-ordered crystals of TBPOSS on water surface. The results of this study may help us to understand the recent evidences of metastable amorphous-to-crystalline reversible phase transformations in inorganic materials like GaSb^6^ and also reversible martensitic transitions in some shape-memory alloys, which have been actively considered as medical sensors and actuators, eco-friendly refrigerators, and energy conversion devices^7^,^8^. There are also reports on reversible shape changes of molecular crystals which find widespread usage as actuators under the influence of external stimuli like photo-irradiation^9^.The general consensus is that for such macroscopically reversible transitions, the symmetry groups of the initial and the final phases are of paramount importance^8^. To the best of our knowledge the quantitative structural evaluation of reversible phase transitions to form bulk-like crystals from monolayers reported here, has not been reported earlier.

Polyhedral Oligomeric SilSesquioxane (POSS) molecules possess a cage-like structure (1–3 nm in size) and a hybrid chemical composition (RSiO_1.5_)*_n_* which is intermediate between silica (SiO_2_) and silicones (R_2_SiO). R can be hydrogen, an alkyl, alkylene, aryl, or arylene group, or an organofunctional derivative of these latter groups and *n* is an even number^10^. Their well defined structures contain a stable inorganic Si-O core surrounded by groups which can be chemically tailored to include a wide range of polarities and reactivity^11^,^12^. This unique property has accelerated potential applications in space-survivable coatings^13^, shape memory materials^14^, semiconducting polymers^15^ and as synthetic templates for nanostructured materials^16^. POSS molecules can be incorporated into polymer systems through blending, grafting or copolymerization and can be used as technologically important nano-composite polymeric materials for reinforcement^17^,^18^ whose properties can be tuned between organic plastics and ceramics. Such techniques have successfully improved physical and chemical properties such as super-oleophobicity^19^, increased glass-transition temperatures^20^, low-k dielectric constants^21^ and thermal stability^22^.

There has been considerable interest to improve on the mechanical stability, swelling behaviour and thermo-mechanical properties of hydrogels, which act as host for the POSS molecule, and has led to extensive research work over the last couple of decades. Enhancement of such properties to more than 300% of the original by adding small amounts of POSS (<5%) have been reported^23^. The augmentation in mechanical properties of hybrid materials urges potential usage in high quality plastics and rubber.

Apart from technological applications, POSS monolayer on water can be a very important system to understand two-dimensional ordering in the presence of hydrophilic and hydrophobic interaction as the functional groups of POSS can be tuned by appropriate chemistry to change its affinity towards water. In case of TBPOSS the Si-O-cage has both hydrophobic (-R = -CH_2_CH(CH_3_)_2_) as well as hydrophilic (-OH) functional groups (see Fig. 1(a)) rendering an amphiphilic property to the molecules. Functionalized POSS monolayers undergo structural transition when compressed in a Langmuir trough^24^,^25^,^26^ and consequently show interesting changes in their viscoelastic response and surface rheology^27^,^28^. However, the structural phase transitions (bringing about appreciable in-plane as well as out-of-plane restructuring) in the monolayer of the POSS itself, when compressed on a Langmuir trough has not been investigated in details. Having an insight into the structural behaviour (pre and post phase-transition) should in principle be very useful for understanding the ordering and restructuring of the molecules and provide viable routes for fabrication of ultra-thin films of POSS with fascinating physical and chemical properties.

In this paper, we report results of an *in-situ* Grazing Incidence X-ray Scattering (GIXS) study that exhibits reversible process of formation of a crystalline phase of TBPOSS from a monolayer phase on water surface. We evidence that under compression, the GIXS patterns exhibit very clear changes going from the existence of a single correlation peak (characteristics of a monolayer on water surface) to powder Debye-Scherrer rings and finally to intense well defined Bragg spots which are oriented with respect to the water surface. The most intriguing result is the reversibility in formation of such patterns under repeated compression-decompression cycles showing the appearance and disappearance of crystalline order as per the surface pressure imposed by pressure barrier of the Langmuir trough. We have proposed here an explanation of the observed reversible monolayer-to -crystalline phase transition based on dimerization of molecules following an earlier study^25^. Based on Brewster angle microscopy measurements of the TBPOSS monolayer at air water interface, it was proposed^25^ that in a collapsed phase, dimers form in the upper layer by hydrogen bonding. The experimental results observed here can be explained assuming that the dimers having hydrophobic shells, arrange in crystalline stacks upon compression on the water surface and relax back to the monolayer phase upon withdrawal of lateral pressure. We also observed that the crystal structure of TBPOSS on water surface differed from the one observed in solid form by conventional powder diffraction^29^. The results are supported by Atomic Force Microscopy (AFM) measurements on films which were transferred to solid substrates both before and after the formation of TBPOSS crystals. Sizes of the crystallites obtained from GIXS measurements on the film post transition matched well with the dimensions of the crystallites observed through AFM on the film transferred to the substrate.

## Experimental

A custom-built Teflon trough including a surface tensiometer (to measure surface pressure), controlled by a NIMA controller box (NIMA, UK) and interfaced with a computer data acquisition system was used for all experiments. TBPOSS (from Hybrid Plastics, USA) was dispersed in Toluene and an aliquot with a concentration of 0.3 mg/mL was prepared. About 80 *μ*L of the solution was spread carefully using a micro-syringe on the trough mounted on an anti-vibration device over the liquid spectrometer and left undisturbed for an hour for the toluene to evaporate. The single barrier trough was contained in an air-tight box with Kapton windows at opposite ends for access to incident and scattered x-ray beam. The trough was purged with Helium gas until the oxygen level in the trough environment decreased to about 1% to ensure the removal of residual vapours of toluene as well as reduce air scattering.

**Figure 1:**
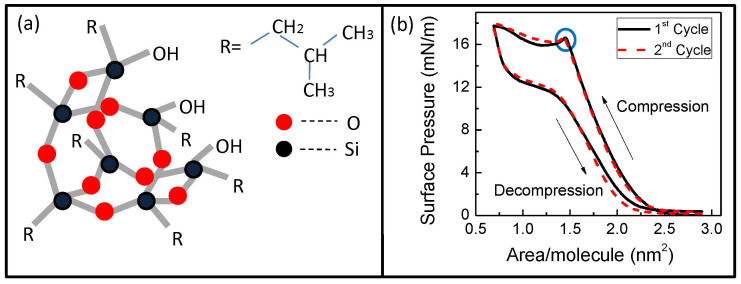
(a) Schematic molecular structure of TBPOSS, here –R denotes –isobutyl group. (b) Pressure-Area isotherm of TBPOSS monolayer on water, recorded at 20°C for 1^st^ (black solid line) and 2^nd^ (dashed red line) compression-decompression cycles. The repeatability of the isotherms shows that the monolayer phase is retained even after undergoing a phase transition at each cycle at the same pressure and area per molecule. Blue circle denotes the transition pressure which is same for the 1^st^ as well as the 2^nd^ cycle.

The monolayer was compressed at a speed of 10 mm/min at a temperature of 20°C to record the Pressure-Area isotherm. The surface pressure rose steadily on regulated compression of the molecules from the gaseous state to a condensed state and a phase transition was observed around 1.45 nm^2^ area per molecule for the surface pressure of 17 mN/m (see Fig. 1(b)). The pressure reduced slightly immediately after the transition but remained constant throughout even for further compression. When the monolayer was decompressed the pressure fell sharply to12 mN/m, remained almost constant for a while and then decreased to zero. In the second cycle we could observe the same behaviour of the monolayer with almost no material loss. The transition pressure was exactly at the same area per molecule (≈1.45 nm^2^) and pressure (≈17 mN/m) even for the second and consequent cycles (Fig. 1(b)). This suggests the TBPOSS molecules form a rugged monolayer on the water surface due to their amphiphilic nature and are highly reproducible over repeated isotherm cycles. The flat portion in the isotherm is indicative of a dynamical state of molecular stacking which is primarily responsible for the intensity fluctuation observed since the molecules move in and out of the area irradiated by the x-ray beam.

**Figure 2:**
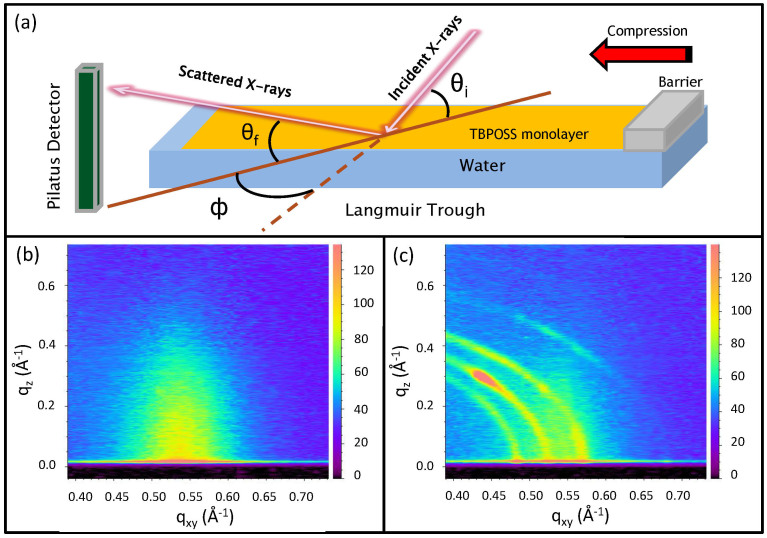
(a) Geometry of the scattering experiment on a liquid spectrometer having a Langmuir trough. Red arrow shows direction of compression. Grazing Incidence X-ray Scattering (GIXS) data from monolayer of TBPOSS on water surface (b) before phase transition showing a inter-particle correlation peak at q_xy_ = 0.54 Å^−1^ and (c) post-transition having defined Scherrer rings/arcs (corresponding to definite crystalline planes) along with bright spots indicative of preferred orientation of the reflecting planes. The presence of a weak correlation peak in the background of the Scherrer rings signifies the presence of a reduced area of monolayer underneath the crystalline stacking. Intensity scales have been provided alongside plots.

X-ray scattering measurements (see Fig. 2(a) for schematics of experimental geometry) were performed on the monolayer both before and after the transition to check the in-plane as well as out-of-plane ordering of the monolayer. Grazing Incidence X-ray Scattering (GIXS) measurements were performed at ChemMatCARS, Sector 15, Advanced Photon Source, Argonne National Laboratory (USA) using 10 keV (*λ* = 1.24 Å) x-ray beam with fixed incident angle *θ_i_* (= 0.1°) which is below the critical angle of water (0.123°) at this energy. A low angle of incidence (lower than the critical angle of water) ensured a low penetration depth of the impinging x-rays due to which the scattering information obtained was mainly from the monolayer. The incident x-ray beam was defined by incident slits of dimension 40 *μ*m × 200 *μ*m (Vertical × Horizontal). The scattered x-rays were detected using a Pilatus 100 K Detector (Dectris) with pixel size of 172 × 172 *μ*m^2^ and only a horizontal detector slit of 300 *μ*m in front of it. The scattered intensities were then plotted as function of in-plane and out-of-plane components (q_xy_, q_z_) of the momentum transfer vector (**q**) which is related to the in-plane angle (*φ*) and out-of-plane angle (*θ_f_*) respectively^30^,^31^,^32^,^33^. This particular detector-slit configuration facilitated the detection of scattered data as a function of *θ_f_*, similar to 1D position sensitive detector (PSD) as shown in Fig. 2(a)^34^. The two-dimensional (2D) GIXS patterns were obtained by scanning the detector along *φ*. The intensity distribution along q_xy_ and q_z_ provides substantial information about the in-plane and out-of-plane structures^31^,^32^,^33^ respectively.

X-ray Reflectivity (XRR) and Grazing Incidence X-ray Off-Specular (GIXOS) measurements were also performed but were inconclusive about any out-of-plane structure due to a lack of electron density contrast between TBPOSS and water. Prior to the phase transition, GIXS measurements showed a characteristic peak around a q_xy_ value of 0.54 Å^−1^ (Fig. 2(b)) which is attributed to short range in-plane inter-particle correlations. After the phase transition the q_xy_ region around the correlation peak transformed into well defined arcs resembling powder diffraction patterns (Fig. 2(c)) along with the weak correlation peak signifying the presence of a monolayer reduced in area due to the compression and subsequent molecular stacking on top of it. Intense spots on the arcs tend to appear and disappear intermittently. This is a clear signature that some preferred orientation of the crystalline planes occurs as a result of some movement of the crystallites over water surface within the coherent scattering volume despite the fact that during the process of data acquisition the barriers were locked after having reached the desired surface pressure. The x-ray data were collected carefully for short duration of time and regular change of sampling area was done to avoid beam-damage^33^. AFM measurements (Nanoscope IV, Digital Instruments) were carried out in tapping mode using etched Si cantilever on thin films of TBPOSS (pre and post phase transition) after transferring horizontally on hydrophilized Si substrate.

## Results and Discussion

The results of the GIXS measurements presented here clearly show that a reversible monolayer-to-crystalline phase transition occurs at 1.45 nm^2^ area per molecule for the surface pressure of 17 mN/m (refer Fig. 2). The GIXS contour in Fig. 2(b) shows an inter-particle correlation peak at q_xy_ = 0.54 Å^−1^ for the monolayer phase. This provides an estimate of the in-plane inter-particle separation between the TBPOSS molecules and corresponds to a length scale of 11.5 Å which is quite close to the molecular dimension of TBPOSS. The values of the in-plane scattered angles (*φ* = 2θ) for the crystalline phase were estimated from the intensity profiles of the GIXS plot (after transition) at q_z_ = 0 (Fig. 2(c)) and the corresponding d_hkl_ values were determined by fitting the arcs assuming a triclinic crystal structure with 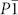 symmetry. The values of 2θ (or d_hkl_) thus obtained were used to determine the following lattice parameters: a = 13.824 Å, b = 15.481 Å, c = 14.015 Å, α = 88.57°, β = 110.41° and γ = 109.51°. A decent agreement between the calculated and observed d_hkl_ values (see Table 1) is found but some of the planes corresponding to calculated values for a particular set of h, k, l values appeared to be missing in the data. Note that with only 5 reflections measured with the available detector, the uniqueness of this set of parameters is not clear. For this reason we have tried to find a set of parameters as close as possible to the ones already published from powder diffraction^29^, i.e., a = 14.697 Å, b = 14.816 Å, c = 15.667 Å, α = 67.70°, β = 67.89° and γ = 71.14°. In this respect, the volume of the unit cell of the crystals forming on water surface is 2635 Å^3^ while for bulk crystal cited in literature it is 2858 Å^3^. This shows that the crystals forming on water surface are constricted in volume with respect to bulk crystals precipitated from solution^29^. A possible explanation for this contraction of the unit cell volume can be found in the fact that reversibility demands non-equilibrium structure of the amphiphilic TBPOSS and the proximity with water strains the unit cell dimensions of the molecules when compared to the bulk crystalline state. The formation of dimers constituting two TBPOSS molecules^29^ is due to the hydrophobic nature of the alkyl arms and the hydrophilic nature of the –OH groups.

**Figure 3:**
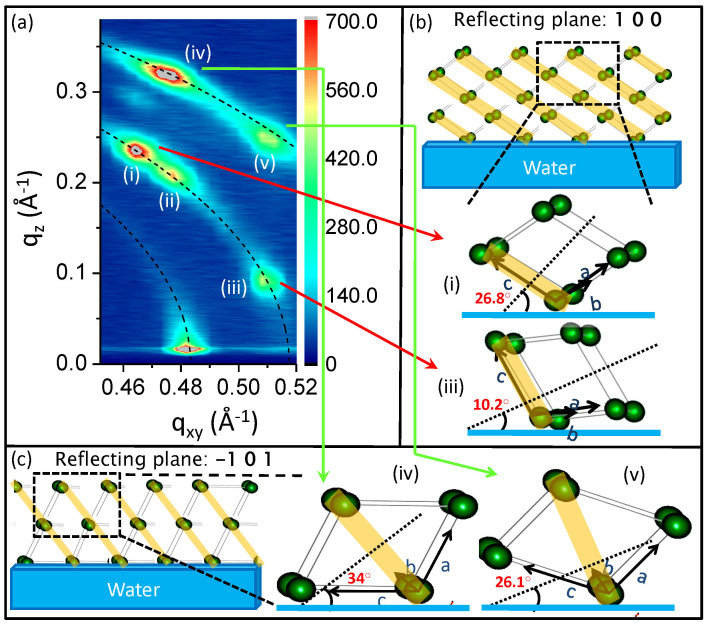
(a) GIXS measurements from monolayer of TBPOSS on water surface and the preferred angular orientation of the TBPOSS dimers for different planes. The dotted lines are fits corresponding to different d_hkl_ values. (b) (i) and (iii) show two angles (26.8° and 10.2°) at which the dimers (shown within border of dashed box for only one angle) are tilted and correspond to the spots designated as (i) and (iii) respectively in the GIXS plot for the (1 0 0) reflecting plane. The respective reflecting planes are shown in yellow shades. (c) (iv) and (v) show similar two angles (34° and 26.1°) of the dimer tilt angle corresponding to the spots (designated as (iv) and (v) in GIXS plot) for the (−1 0 1) plane.

**Figure 4:**
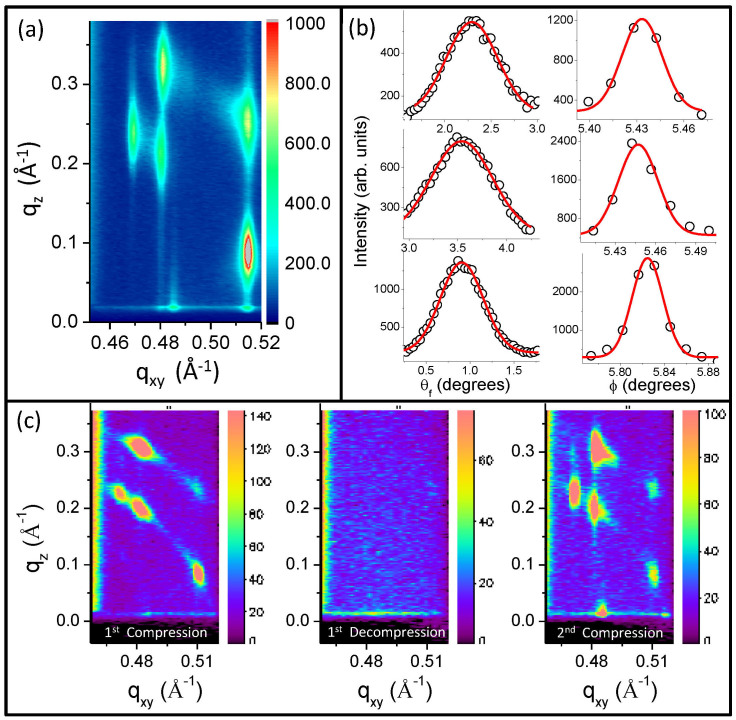
(a): GIXS measurements on the monolayer, after transition in the 2^nd^ cycle, reveal the onset of 2D ordering in the monolayer (on top of which the dimers form columnar stacks) due to the appearance of rods in place of spots. (b) Intensity profiles (scattered plots) along the rods (θ_f_ or q_z_) and across the rods (φ or q_xy_) and respective Gaussian fits (red lines) to ascertain the FWHM from which the in-plane as well as out-of-plane crystal grain size is estimated using Scherrer formula. (c): GIXS measurements showing spots on arcs after phase transition for 1^st^ compression cycle, no spots or arcs for 1^st^ decompression cycle and spots replaced by rods after another phase transition (at the same pressure) for the 2^nd^ compression cycle.

The presence of intense spots at different positions on the Debye Scherrer ring upon compression is a clear signature that the system evolves towards crystallites that are preferentially oriented with respect to the water surface (Fig. 3(a)). Two of the several planes (viz. 1 0 0 & −1 0 1) were more intense than the others and were considered for further analysis. The preferred tilt angles of the dimer planes with respect to the water surface were estimated directly from the position of the spots in the GIXS profile (Fig. 3) and have been tabulated in Table 2. The crystallite size obtained from GIXS measurements is estimated by the Scherrer formula given by,
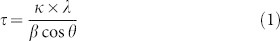
where τ is the crystal grain size, κ is a constant (0.94), λ is the wavelength (1.24 Å), θ is half the angle corresponding to the intensity peak and β is the FWHM of the peak around 2θ. The values were calculated for the five prominent rods (Fig. 4(a)) by estimating the FWHM (three representative profiles are shown in Fig. 4(b)) along the rods and perpendicular to the rods. The average crystal grain size comes out to be approximately2000 Å in the in-plane direction and 100 Å in the out-of-plane direction.

The isotherm was repeated and the second cycle closely followed the first. The characteristic phase transition was also observed to occur at the same pressure for the second cycle (Fig. 1(b)) but the GIXS measurements showed that the bright spots over defined arcs obtained in the first isotherm cycle (Fig. 3(a)) were replaced by Bragg rods with almost no arcs at all in the second isotherm cycle (Fig. 4(a)). Interestingly, the positions of the rods coincide with those of the spots obtained in the first isotherm cycle. The preferred orientation of the planes is responsible for the observed spots on the Scherrer rings for 1^st^ cycle and rods for subsequent cycles. The presence of the spots in the 1^st^ cycle is primarily due to the variance in the orientation of the favoured crystal planes and additionally due to positional uncertainty of the relatively loosely stacked motifs within the crystals. However, no spots or rods were observed when the monolayer was decompressed. The rods were also short-lived like their predecessors. This is a clear signature of increase in the in-plane correlation length. It appears that over repeated compression-decompression cycles, 2D ordering commences in the monolayer after the first cycle. The sequence of events depicted in Fig. 4(c) show clear spots over arcs in the GIXS data after phase transition in the 1^st^ cycle, no features are visible when the monolayer is decompressed and spots are replaced by rods after phase transition in the 2^nd^ cycle.

**Figure 5:**
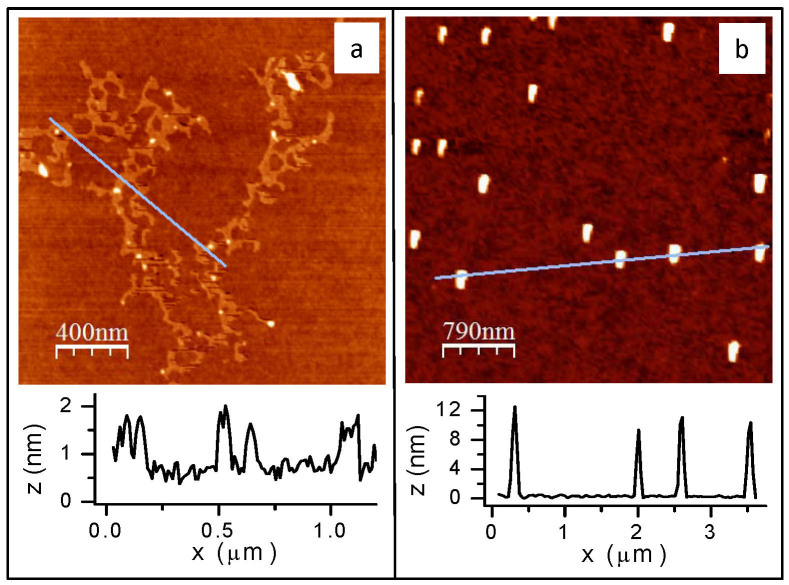
AFM images of the thin films of TBPOSS transferred on Si substrate from water surface, pre and post transition. (a) shows dendritic network of POSS monolayer of average thickness 12 Å and (b) shows crystal grains of average size ~2004 Å and thickness ~101 Å. The crystal grain size from AFM measurements compares remarkably well with that estimated from the GIXS data using the Scherrer formula.

The AFM topographs revealed presence of a dendritic monolayer of TBPOSS (height 12 Å) for the sample transferred before phase transition (see Fig. 5(a)) and presence of crystallites of average lateral size and height of 2004 Å and 101 Å, respectively, for the sample transferred after transition (see Fig. 5(b)). The height of the TBPOSS monolayer (12 Å) obtained from AFM is in agreement with the in-plane inter-particle distance extracted from GIXS measurements (11.5 Å) for the film before transition. The average lateral dimensions and the height of the crystallites seen in the film post transition through AFM match well with the in-plane and out-of-plane dimensions of the crystallites obtained from GIXS data.

**Figure 6:**
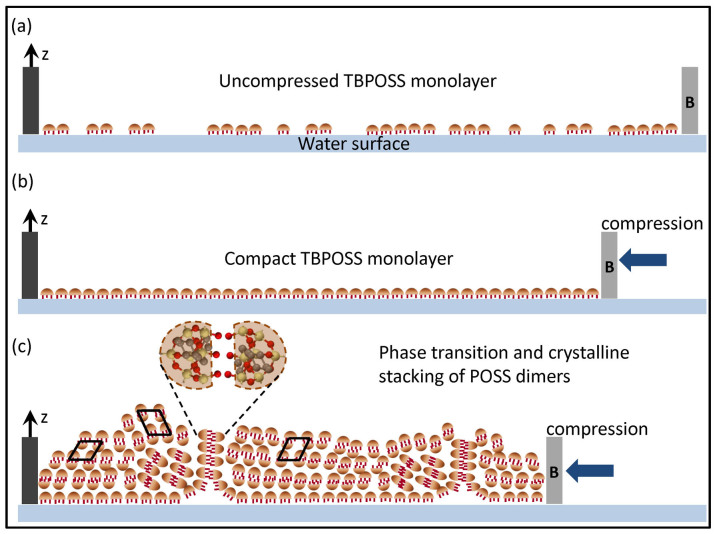
Schematic illustration of the mechanism of reversible meta-stable transition of TBPOSS molecules from monolayer phase to crystalline phase by stacking of molecular dimers as a function of lateral pressure. The movable barrier is denoted by B. (a) TBPOSS monolayer before compression and (b) after 1^st^ compression which leads to compact monolayer resulting in an inter-particle correlation peak in the GIXS profile. (c) Further compression leads to a phase transition to crystalline phases wherein the molecular dimers form crystalline stacks over the monolayer. The preferential alignment of the stacking planes with respect to the water surface over the monolayer is responsible for the intense spots on the Scherrer rings. These spots are replaced by intense Bragg rods on further compression-decompression cycles.

Based on detailed analysis of x-ray scattering data collected before and after the transition from a monolayer to non-equilibrium crystal phase, we now propose a mechanism of reversible phase transition observed here (shown schematically in Fig. 6(a)–(c)). In this proposed mechanism it is assumed that the monolayer-to-crystal phase transition is preceded by dimer formation. It is to be noted here that the concept of such dimers in TBPOSS was initially proposed^29^ on the basis of x-ray power diffraction measurements and was also used to explain the collapse of a Langmuir monolayer^25^. We believe that once the dimers form, they spontaneously self-assemble into non-equilibrium crystal structures on further compression. This gives rise to the sharp transition peak in the isotherm but only one peak is visible in the isotherm upon compression as reported earlier^25^. Below this transition peak during compression we could only measure a correlation peak in the GIXS measurements while above this peak, at lower area per molecule, the GIXS suddenly showed crystalline diffraction peaks. This is a signature of the transition from a monolayer to a dimer and then eventually to the crystalline phase as a kinetically driven process. It was not possible in this experiment to evidence the transient regime from a dimer to crystalline phase - future studies using a nano-sized x-ray beam utilizing detectors with much faster data acquisition capability may be able to decipher this kinetically driven phase transition.

The dimers start forming unstable crystals primarily by van der Waals forces when compressed. On decompression, the dimers which were stacked together as crystallites came in contact with water and the crystalline stacking disappeared. The monolayer phase was re-established but the dimers did not completely disintegrate into monomers. Further compression cycles forced these aligned dimers into a more compact packing leading to better alignment of the crystals in 2D than formed during the first compression cycles thus justifying the existence of Bragg rods (onset of 2D ordering in the monolayer) instead of spots in further compression/decompression cycles. This repetitive process of crystalline stacking and unstacking of the molecules as a function of lateral compression clearly supports the concept of meta-stable crystal formation as schematically illustrated in Fig. 6. The crystal structure so formed has certain preferential orientations with respect to the water surface. The reason for these preferential orientations is still not completely understood. Similar studies with other POSS molecules with different end groups attached have to be performed to understand the nature of monolayer-to-crystalline phase reversibility.

## Summary and Conclusion

Characteristic phase transition has been observed for TBPOSS monolayer on water surface. The isotherms in consequent cycles were reversible and in each cycle the phase transition occurred at the same area per molecule and pressure as previous cycles. GIXS measurements have been performed on monolayer of TBPOSS before and after transition. GIXS data show that the TBPOSS monolayer exhibits a rare reversible non-equilibrium crystalline phase on lateral compression. The crystal structure obtained here using GIXS measurements is different from the one obtained by conventional powder diffraction measurements for TBPOSS^29^. Although the crystal structure and symmetry were the same as reported, we observed a constricted lattice for the triclinic structure. GIXS measurements are consistent with AFM measurements done on films transferred both before and after the phase transition. We can subsume that the crystalline phases observed due to application of lateral pressure are apparently reversible meta-stable phases formed by stacking of molecular dimers which relax to the initial monolayer phase as soon as the lateral pressure is removed. The detailed understanding of the mechanism of stacking and relaxing of the molecular dimers as a function of lateral pressure necessitates further investigation by altering the alkyl and the hydroxyl groups of the POSS molecule. This phenomenology can be extended to explain the process of transient reversible crystallization observed in nature in various other materials.

## Author Contributions

R.B., M.K.S. and M.K.B. designed and performed the experiment and did the data analysis, B.L. and M.M. supported the experiments and data analysis, A.G. provided useful discussions and performed additional theoretical calculations. The paper was written by R.B., M.K.S., M.K.B. and A.G. and edited by all authors.

## Figures and Tables

**Table 1:** The observed* and calculated values corresponding to different d_hkl_ planes have been tabulated. Some of the Bragg planes were missing in the observed GIXS data. Lattice parameters and space group used to calculate the (h k l) values have been discussed in the text

Observed d_hkl_ (Å)	Calculated d_hkl_ (Å)	Calculated (hkl) values
-	14.5138	0 −1 0
		0 1 0
13.0650	13.0648	0 0 1
12.1500	12.1502	−1 0 0
		1 0 0
11.4833	11.4831	1 −1 0
		−1 1 0
11.1242	11.1242	−1 0 1
-	10.2527	0 −1 1
-	10.0083	−1 1 1
9.2458	9.2560	0 1 1

^*^All measurements were recorded at 20°C.

**Table 2:** The values of the preferred angles for different reflecting planes obtained from the GIXS contour. Labels correspond to spots as in Fig. 3

Plane	Labels	Angles (°)
1 0 0	(i)	26.8
	(ii)	23.4
	(iii)	10.2
−1 0 1	(iv)	34
	(v)	26.1
